# Abiraterone in patients with recurrent epithelial ovarian cancer: principal results of the phase II Cancer of the Ovary Abiraterone (CORAL) trial (CRUK – A16037)

**DOI:** 10.1177/1758835920975352

**Published:** 2020-12-29

**Authors:** Susana Banerjee, Holly Tovey, Rebecca Bowen, Elizabeth Folkerd, Lucy Kilburn, Jennifer McLachlan, Marcia Hall, Nina Tunariu, Ayoma Attygalle, Joao Paulo Da Silveira Nogueira Lima, Sophie Perry, Peter Chatfield, Margaret Hills, Stan Kaye, Gert Attard, Mitch Dowsett, Judith M. Bliss

**Affiliations:** Gynaecology Unit, The Royal Marsden NHS Foundation Trust, Downs Road, Sutton, SM2 5PT, UK; The Institute of Cancer Research, London, UK; Clinical Trials and Statistics Unit, The Institute of Cancer Research, London, UK; Royal United Hospitals Bath NHS Foundation Trust, Bath, UK; The Institute of Cancer Research, London, UK; Clinical Trials and Statistics Unit, The Institute of Cancer Research, London, UK; Department of Medical Oncology, Christchurch Hospital, Christchurch, New Zealand; Medical Oncology, Mount Vernon Cancer Centre, Northwood, UK; Radiology, The Royal Marsden Hospital NHS Foundation Trust, London, UK; Histopathology, The Royal Marsden Hospital NHS Foundation Trust, London, UK; A.C. Camargo Cancer Center, Oncologia Clinica, São Paulo, Brazil; Clinical Trials and Statistics Unit, The Institute of Cancer Research, London, UK; Clinical Trials and Statistics Unit, The Institute of Cancer Research, London, UK; The Royal Marsden Hospital NHS Foundation Trust, London, UK; Gynaecology Unit, The Royal Marsden NHS Foundation Trust, London, UK; UCL Cancer Institute, University College London, London, UK; The Royal Marsden NHS Foundation Trust, Sutton, UK; Clinical Trials and Statistics Unit, The Institute of Cancer Research, London, UK

**Keywords:** abiraterone, androgen receptor, CYP17 inhibitor, low grade serous, ovarian cancer

## Abstract

**Background:**

Recurrent epithelial ovarian cancer (EOC) remains difficult to treat, with an urgent need for more therapy options. Androgens bind to the androgen receptor (AR), commonly expressed in EOC. CYP17 inhibitor abiraterone irreversibly inhibits androgen biosynthesis. The Cancer of the Ovary Abiraterone (CORAL) trial was designed to evaluate the clinical activity of abiraterone in EOC.

**Patients & Methods:**

CORAL was a multi-centre, open-label, non-randomised, 2-stage phase II clinical trial. Eligible patients had progression within 12 months of last systemic therapy and no prior hormonal anti-cancer agents. Patients received abiraterone 1000 mg daily plus 5 mg prednisone until progression. The primary endpoint was objective response rate (ORR) according to combined Response Evaluation Criteria in Solid Tumours/Gynaecological Cancer Intergroup (RECIST/GCIG) criteria at 12 weeks. Secondary endpoints included clinical benefit rate (CBR) at 12 weeks.

**Results:**

A total of 42 patients were recruited; median age 65 (range 34–85) years; 37 (88.1%) had high-grade serous tumours; 20 (48%) had at least three prior lines of therapy; 29/40 (72.5%) were AR+. In stage 1, 1/26 response was observed (in an AR+, low-grade serous EOC); response lasted 47 weeks. Overall, 12 week ORR was 1/42 (2%), CBR was 11/42 (26%) (8/29 (28%) in AR+ patients). Disease control was ⩾6 months for 4/29 (14%). One patient (AR+, low-grade serous) had a RECIST response at 82 weeks. Four (10%) had grade ⩾3 hypokalaemia; 11 (26%) had dose delays.

**Conclusions:**

CORAL represents the first trial of an AR targeted agent in ovarian cancer. While responses were rare, a subset of patients achieved sustained clinical benefit. Targeting AR in EOC including low-grade serous cancer warrants further investigation.

**Trial registration:**

CORAL is registered on the ISRCTN registry: ISRCTN63407050; http://www.isrctn.com/ISRCTN63407050

## Introduction

Approximately 75% of women with epithelial ovarian cancer (EOC) present with advanced [International Federation of Gynaecology and Obstetrics (FIGO) stage III/IV] disease. Despite high response rates to primary surgery and chemotherapy, the majority of women with advanced disease ultimately relapse, and subsequently die from progressive disease. It is hypothesised that individualisation of treatments based on molecular markers that characterise the tumour biology will lead to successful treatment strategies for advanced EOC.^1^

Currently, no hormonal therapy has a licensed indication for EOC. Although over 70% of EOC express oestrogen (ER) and/or progesterone (PGR) receptors, treatment with tamoxifen, aromatase inhibitors or luteinising hormone releasing hormone (LHRH) agonists has shown minimal activity. A Cochrane Review of tamoxifen activity in relapsed ovarian cancer demonstrated an overall response rate of 10% and stable disease for ⩾4 weeks in an additional 32%.^2^ Phase II studies of letrozole similarly reported response rates between 3% and 17%.^3–5^ More recently, a phase II trial of anastrozole in platinum-resistant or refractory ovarian cancer reported a clinical benefit rate of 27%; however, no patients achieved a documented response.^6^

The androgen receptor (AR) is expressed in up to 90% of EOC cases.^7–10^ Co-activators, such as AR-associated protein 70, AIB1 and p44 have been reported to influence AR activity in ovarian cancer.^11–13^ AR may influence epidermal growth factor receptor (*EGFR)* and transforming growth factor-beta (TGFβ) signalling in ovarian cancer affecting tumour growth.^9,14,15^ Preclinical models of ovarian cancer have demonstrated cellular proliferation with androgenic stimulation correlating with AR expression.^16^ However, the activity of anti-androgens tested so far in EOC is limited.^9^ Phase II studies of cyproterone and flutamide reported response rates of 4–7%.^17–19^ A study of goserelin and bicalutamide in second or higher clinical remission reported a progression-free survival (PFS) of 11 months.^20^ Androgen signalling pathway inhibitors, (e.g. abiraterone, enzalutamide), which have shown success in prostate cancer, have the potential for clinical activity in ovarian cancer. In preclinical studies, enzalutamide, an AR inhibitor, was shown to significantly reduce growth of EOC xenografts,^21^ providing support for exploring this treatment approach.

Abiraterone acetate, a prodrug of abiraterone, is a novel cytochrome P450 c17 (CYP17) inhibitor that irreversibly inhibits generation of adrenal steroids downstream of CYP17. Abiraterone in combination with prednisone is approved for use in men with metastatic castration-resistant prostate cancer and also in newly diagnosed high risk metastatic hormone sensitive prostate cancer, with phase III studies demonstrating an overall survival EN(OS) benefit and an acceptable toxicity profile.^22–24^ Phase II trials of abiraterone in ER positive breast cancer and AR positive triple-negative breast cancer have been reported minimal activity.^25,26^ To date, there have been no studies of abiraterone in EOC.

The aim of the Cancer of the Ovary Abiraterone Trial (CORAL) was to assess the activity and safety of abiraterone acetate plus prednisone in hormone-treatment naive patients with recurrent epithelial ovarian, fallopian tube or primary peritoneal cancer.

## Methods

### Trial design and patient population

CORAL (ISRCTN63407050; CRUK A16037) was a prospective, multi-centre, open-label, non-randomised, two-stage phase II clinical trial conducted in three United Kingdom (UK) centres. Post-menopausal women with histologically or cytologically confirmed epithelial ovarian, fallopian tube (FT) or primary peritoneal (PP) cancer were eligible if they had progressed (radiological or CA125 criteria) within 12 months of last systemic anti-cancer therapy. Additional inclusion criteria included disease measurable by Response Evaluation Criteria in Solid Tumours (RECIST; version 1.1) or evaluable by Gynaecological Cancer Intergroup (GCIG) CA-125 criteria; at least one line of prior platinum-based chemotherapy and no prior hormone therapy. Patients with mucinous, clear cell, malignant mixed mesodermal or non-epithelial ovarian histologies were excluded (see supplemental appendix for full eligibility criteria).

CORAL was approved by the Medicines and Healthcare products Regulatory Authority (MHRA) and the London–Westminster Research Ethics Committee (REC 13/LO/1599). All enrolled patients provided written informed consent.

### Treatment allocation and study procedures

Abiraterone acetate was prescribed at a dose of 1000 mg orally once daily in a continuous 28 day cycle until disease progression by RECIST version 1.1 or death. Prednisone or prednisolone (at clinician’s discretion) was started at 5 mg orally once daily to prevent secondary mineralocorticoid excess. Dose modifications were required for any grade 3 or 4 toxicity considered possibly related to abiraterone. A dose increase for prednisone/prednisolone of up to 10 mg/day (5 mg twice daily) was permitted to manage mineralocorticoid-related toxicities.

Radiological assessment by computed tomography (CT)/magnetic resonance imaging (MRI) scan was performed at baseline and every 12 weeks until progression. Adverse events were graded according to National Cancer Institute (NCI) Common Terminology Criteria (CTC) version 4.0. The primary endpoint was objective tumour response rate at 12 weeks defined as the proportion of patients with complete or partial response at 12 weeks from registration by combined RECIST/GCIG CA125 criteria. Patients who discontinued prior to 12 weeks were classed as non-responders. Secondary endpoints defined using RECIST/GCIG CA125 as above included clinical benefit rate (CBR: proportion of patients with complete/partial response or stable disease at 12 weeks), PFS, 6-month PFS rate, OS; tolerability and safety.

AR, Ki67, ER, PGR and HER2 were evaluated on archival tumour tissue. Plasma levels of oestradiol, testosterone, dehydroepiandrosterone (DHEAS), androstenedione and corticosterone were measured at baseline, cycle 2 day 1, cycle 4 day 1 and progression (see supplemental appendix for details of antibodies used).

### Statistical considerations

**Figure 1.**
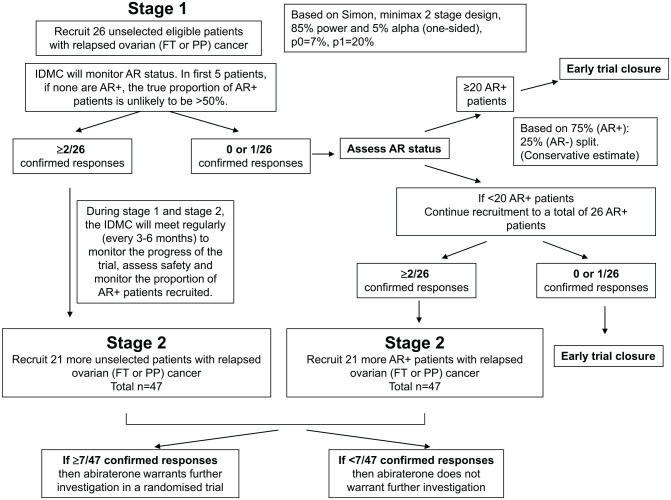
Trial design: Simon 2-stage minimax design, initially in unselected patients with the option to recruit only AR+ patients in stage 2. AR, androgen receptor; FT, fallopian tube; IDMC, independent data monitoring committee; PP, primary peritoneal.

Using a Simon Minimax Two-Stage design this study was designed with 85% power and a one-sided 5% significance level to discount an ‘ineffective’ response rate of 7% (p0) in patients treated with abiraterone in favour of a response rate of 20% (p1) (Figure 1); 26 patients were required in stage 1 with a further 21 in stage 2 if at least 2 responses were observed in stage 1. There was no planned stopping in recruitment between stages 1 and 2. Interim data was reviewed by the joint Independent Data Monitoring and Steering Committee (IDMSC).

The primary outcome analyses included all patients on an intention to treat (ITT) basis. Estimated response rates are reported with 95% confidence intervals (CIs). Survival endpoints are shown graphically using Kaplan–Meier plots and median time from registration estimated. The following subgroup analyses were pre-planned: AR status [positive (>10% tumour cell nuclei being immunoreactive by IHC) *versus* negative]; ER status [positive (H-score ⩾1) *versus* negative]; histological subtypes (high grade serous *versus* other); number of prior relapses (0/1, 2, 3+).

The safety analyses population was defined as patients who received at least one dose of abiraterone with worst adverse event grade during trial treatment reported. Pre-specified toxicities and any MedDRA coded event with ⩾10% incidence are presented.

This analysis includes all data received and processed by 1 August 2017. All analyses were done in Stata (version 13.1; StataCorp LLC, College Station, TX, USA).

## Results

**Figure 2.**
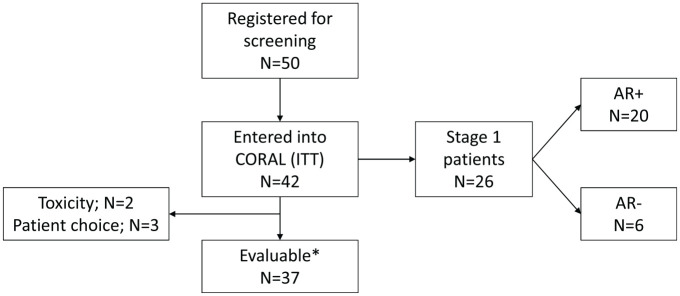
CONSORT flow diagram showing the number of patients entered and evaluable in the trial. *Evaluable defined as completed 12 weeks of treatment or discontinued prior to 12 weeks due to disease. AR, androgen receptor; CORAL, cancer of the ovary abiraterone trial ; ITT, intention to treat.

A total of 42 patients were registered between 21 March 2014 and 3 November 2015. Median follow up in all patients was 30.2 months [interquartile range (IQR): 22.3, 35.0]. In September 2015, the CORAL IDMSC reviewed data for the first 26 patients registered in stage 1; by which time a total of 36 patients had been recruited (Figure 2). Due to insufficient responses, a planned analysis of AR status was required to determine continuation of the trial. The committee advised that recruitment was suspended until the number of AR+ patients could be determined. Six patients who were already undergoing screening were permitted to enter the trial given the absence of safety concerns. In March 2016, the trial formally stopped recruitment (without re-opening) due to insufficient activity observed in AR+ patients.

### Patient demographics and tumour characteristics

The median age at study entry was 65.4 years (IQR 55.7–72.7; range: 34.2–85.2); 39 (92.9%) were FIGO stage III or IV at diagnosis and on average (median) patients had received two prior lines of treatment (range: 1–6 prior lines (Table 1). The median time from first relapse to trial entry was 1.5 years (IQR 1.1–2.6) and from diagnosis to trial entry was 2.8 years (IQR 1.9–4.5). 24 (57.1%) had platinum-resistant/refractory disease and 22 (52.4%) entered the trial less than 12 months from the date of progression that led to the prior line of therapy. A total of 39 (92.9%) patients had RECIST measurable disease and 9 (21.4%) had ascites at baseline; 12 (28.6%) had received prior bevacizumab. The majority of patients, 37 (88.1%), had high grade serous EOC following central review and 3 (7.1%) had low grade serous histology. Hormonal receptor status of tumour tissue was subsequently tested centrally; 29/40 (72.5%) patients were AR+ (>10% staining tumour cells) (two samples unavailable), 35/37 (95%) ER+ and 25/37 (68%) PGR+ (five samples undetermined). No tumours were HER2 positive.

**Table 1.** Baseline patient and tumour characteristics.

		Total (*n* = 42)	
		*n*	*%*
Age (years)	Mean (SD)	64.6 (10.9)	
Age group (years)	30–40	1	2.4
	40–50	2	4.8
	50–60	10	23.8
	60–70	17	40.5
	70–80	9	21.4
	80+	3	7.1
Time from initial diagnosis to trial entry (years)	Median (IQR)	2.8 (1.9–4.5)	
Time from initial diagnosis to trial entry (years)	<1	1	2.4
	1–3	20	47.6
	3–5	13	31.0
	5+	8	19.0
Time from first relapse to trial entry (years)	Median (IQR)	1.5 (1.1–2.6)	
Time from first relapse to trial entry (years)	<1	8	19.0
	1–3	24	57.1
	3–5	3	7.1
	5+	3	7.1
Time from last relapse to trial entry (months)	Median (IQR)	11.2 (8.6–15.6)	
Time from last relapse to trial entry (months)	<3	2	4.8
	3–6	3	7.1
	6–12	17	40.5
	12+	20	47.6
Disease measurable by RECIST	Yes	39	92.9
	No	3	7.1
Ethnicity	White British	37	88.1
	Other white	1	2.4
	Indian	2	4.8
	African	1	2.4
	Unknown	1	2.4
ECOG at screening	0	20	47.6
	1	20	47.6
	2	2	4.8
Histological type (local assessment)	High grade serous	34	81.0
	Low grade serous	3	7.1
	Endometrioid	2	4.8
	High and low grade serous	1	2.4
	High and other	1	2.4
FIGO stage	I A	1	2.4
	II B	1	2.4
	II C	1	2.4
	III	9	21.4
	III A	1	2.4
	III B	4	9.5
	III C	22	52.4
	IV	3	7.1
Grade	Grade 1	1	2.4
	Grade 2	3	7.1
	Grade 3	35	83.3
	Unknown	3	7.1
ER status (local assessment)	Positive	23	54.8
	Negative	2	4.8
	Unknown	17	40.5
ER status (central assessment)	Positive	35	83.3
	Negative	2	4.8
	Unknown	5	11.9
PGR status (central assessment)	Positive	25	59.5
	Negative	12	28.6
	Unknown	5	11.9
Disease history	Primary only	4	9.5
	Primary + 1 relapse	18	42.9
	Primary + 2 relapses	10	23.8
	Primary + 3 relapses	4	9.5
	Primary + 4 relapses	4	9.5
	Primary + 5 relapses	2	4.8

ER, oestrogen receptor; FIGO, international federation of gynaecology and obstetrics; IQR, interquartile range; PGR, progesterone receptor; RECIST, response evaluation criteria in solid tumours; SD, standard deviation.

### Activity

In the stage 1 population, including the first 26 patients recruited, objective response rate (ORR) was 1/26 (3.9%) and CBR was 7/26 (26.9%); 20 (76.9%) patients were determined to have AR+ tumours. As recruitment was not suspended between stage 1 and 2, 26 patients with AR+ tumours had been recruited; ORR within the first 26 AR+ patients was 1/26 (3.9%).

**Figure 3.**
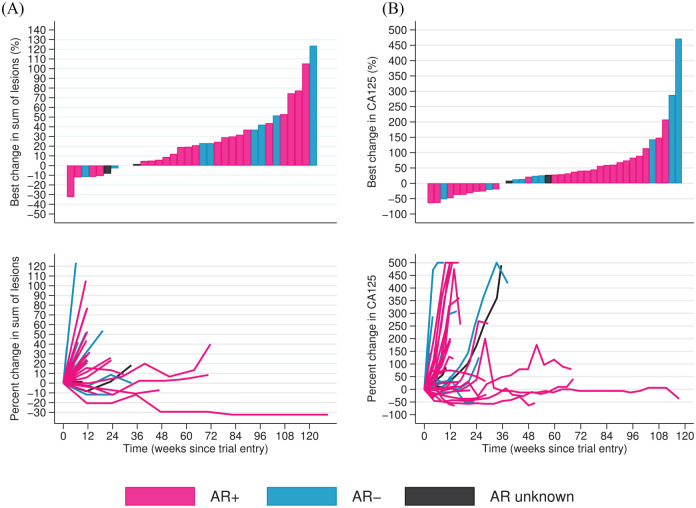
Change in (A) RECIST sum of target lesions and (B) CA125 levels. Waterfall plots showing best percentage change for each patient in (A) sum of target lesions and (B) CA125 levels. Spider plots showing percentage change over time for each patient in (A) RECIST and (B) CA125 levels. Percentage change in CA125 levels capped at +500%. AR, androgen receptor; RECIST, response evaluation criteria in solid tumours.

In the ITT population, one partial response using the combined RECIST/GCIG CA125 criteria was seen at 12 weeks (ORR: 1/42; 2.4%, 95%CI: 0.1, 12.6). The tumour sample of this patient was AR+, ER+ and PGR+; the histological subtype was low grade serous and the response lasted for 47 weeks. This patient with platinum-resistant disease, had received three prior lines of chemotherapy. Her last treatment was liposomal doxorubicin (caelyx) 3 months prior to starting abiraterone. A second patient (low grade serous histology, AR+, ER– and PgR–), had a 29% reduction in target lesions observed at 12 weeks and, after 82 weeks on abiraterone, a 32% reduction in target lesions from baseline was noted amounting to a RECIST partial response (Figure 3).

CBR at 12 weeks was 11/42; 26.2% (95% CI: 13.9, 42.0) and median duration of clinical benefit (in those with clinical benefit at 12 weeks) was 27.1 weeks (IQR = 23.1, 61.6). The CBR rate at 6 months was 5/42; 11.9%.

A total of 41 patients (97.6%) reported a PFS event at the time of analysis; median PFS was 2.5 months (95% CI: 1.8, 3.4) and the PFS rate at 6 months was 16.7% (95% CI: 7.3, 29.3). Median OS was 11.8 months (95% CI: 5.9, 22.1) with 29 deaths reported, all of which were disease related.

### Biological markers/analysis

In subgroup exploratory analyses, no association was observed between AR status, ER status, histological subtype or number of prior lines of therapy and clinical benefit. The ORR rate was 1/29 (3.4%) in AR+ and 0/11 (0.0%) in AR- cases; CBR rate was 8/29 (27.6%) and 2/11 (18.2%) for AR+ and AR– cases respectively (see Table S1). Disease control lasted ⩾6 months in 4 of the AR+ cases who met the criteria for clinical benefit.

**Figure 4.**
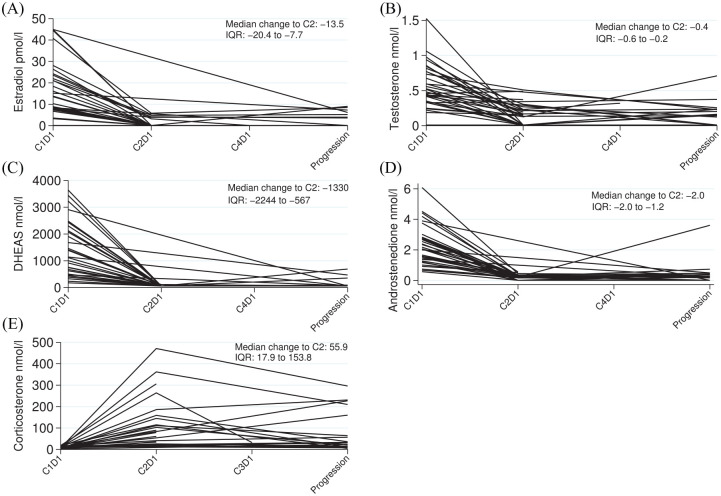
Hormone levels by each patient measured at each visit. Levels of (A) oestradiol, (B) testosterone, (C) DHEAS and (D) androstenedione show reductions following administration of abiraterone. (E) Levels of corticosterone show an initial increase following administration of abiraterone. DHEAS, dehydroepiandrosterone; IQR, interquartile range.

A significant decrease in testosterone, oestrodiol androstendione and DHEAS was observed on treatment at cycle 2 compared with pre-treatment (*p* < 0.001 for all analyses; Figure 4).

### Compliance and tolerability

**Figure 5.**
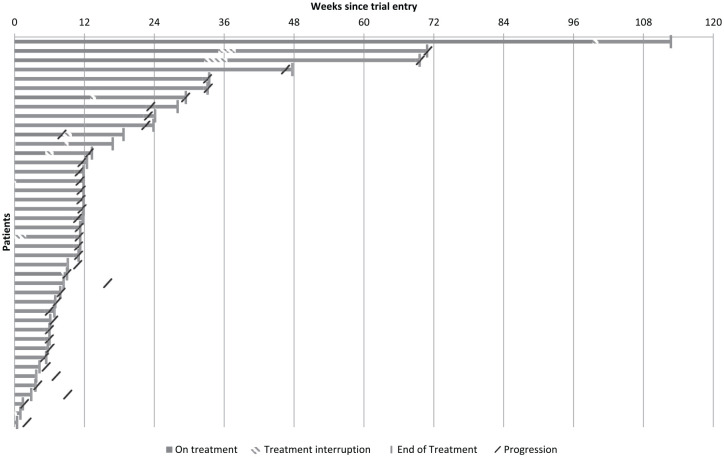
Time on treatment. Number of weeks abiraterone received by each patient illustrating treatment interruptions and discontinuations.

A total of 24 (57.1%) patients received abiraterone until at least the first planned scan assessment. The median time on treatment was 11.3 weeks (IQR 6.0, 18.7) (Figure 5).

The main reason for treatment discontinuation was disease progression (*n* = 32, 76.2%). Three (7.1%) patients discontinued due to toxicity, four (9.5%) due to patient choice, two (4.8%) due to clinician’s decision based on deterioration secondary to disease in the absence of confirmed progression and one (2.4%) patient was unable to attend hospital (unrelated to drug or disease). A total of 11 (23.8%) patients had abiraterone treatment interruption; mean duration of interruption was 8.1 days and was adverse event-related in 85.7% of cases. No dose reductions were reported.

The most common treatment emergent adverse events (any grade and relationship to drug) were abdominal pain (*n* = 24), hypertension (*n* = 21), nausea (*n* = 18), constipation (*n* = 17), fatigue (*n* = 17) and decreased appetite (*n* = 16). The majority of events were grade 1 or 2 and consistent with events related to EOC. Grade 3 hypertension was noted in 28.6% of patients and grade 3 or 4 hypokalaemia was noted in 9.5%. Grade 3 fluid retention occurred in 1 patient (2.4%). There were no deaths related to abiraterone (Table 2).

**Table 2.** Adverse events.

	Adverse event present on treatment		Treatment emergent^a^		Present at grade 3/4 on treatment	
	n	%	n	%	n	%
Abdominal pain	31	73.8	24	57.1	6	14.3
Hypertension	28	66.7	21	50.0	12	28.6
Fatigue	26	61.9	17	40.5	1	2.4
Constipation	22	52.4	17	40.5	0	0.0
Nausea	19	45.2	18	42.9	1	2.4
Decreased appetite	18	42.9	16	38.1	1	2.4
Neuropathy peripheral	16	38.1	9	21.4	0	0.0
Dyspnoea	14	33.3	11	26.2	3	7.1
Vomiting	13	31.0	13	31.0	2	4.8
Back pain	12	28.6	11	26.2	3	7.1
Diarrhoea	11	26.2	11	26.2	1	2.4
Hypokalaemia	11	26.2	10	23.8	4	9.5
Dyspepsia	8	19.0	8	19.0	0	0.0
Abdominal discomfort	7	16.7	7	16.7	0	0.0
Anaemia	7	16.7	7	16.7	0	0.0
Cough	7	16.7	7	16.7	1	2.4
Lower respiratory tract infection	7	16.7	7	16.7	2	4.8
Oedema peripheral	7	16.7	6	14.3	1	2.4
Tachycardia	7	16.7	6	14.3	0	0.0
Hot flush	6	14.3	6	14.3	0	0.0
Urinary tract infection	6	14.3	6	14.3	2	4.8
Hepatic enzyme increased	5	11.9	5	11.9	1	2.4
Headache	4	9.5	4	9.5	0	0.0
Rash	3	7.1	3	7.1	0	0.0
Atrial fibrillation	1	2.4	1	2.4	0	0.0
Glaucoma	1	2.4	1	2.4	0	0.0
Haematuria	1	2.4	1	2.4	0	0.0
Muscle spasms	1	2.4	0	0.0	0	0.0
Muscular weakness	1	2.4	1	2.4	0	0.0

a Treatment emergent is defined as adverse events which were present during treatment but were not present at baseline or which were present at baseline but the grade became worse while on treatment. Adverse events are reported regardless of their relationship to treatment.

## Discussion

CORAL is the first prospective clinical trial of abiraterone in EOC. Given the potential role of AR in the biology of EOC,^9,16^ there was a rationale to assess novel AR signalling therapies as a new targeted strategy. The aim was to establish whether the clinical activity warranted taking forward into a larger, randomised study. The level of activity in the ITT population did not achieve the threshold for continuation into the second part of the two stage phase II trial (2/26 responses required), leading to early trial closure. However, although the desired response rate at 12 weeks was not observed, a subset of patients derived sustained clinical benefit. This trial has provided important information regarding the role of AR-mediated signalling inhibition in patients with recurrent, advanced EOC.

The trial was designed to initially assess the role of abiraterone in a population of EOC patients unselected for AR positivity. The definition of AR positivity varies between clinical studies; thresholds ranging from ⩾1% to ⩾10% nuclear staining have been used.^25,26^ The relevance of the percentage of positive cells and response to AR targeted treatment is not fully known. The mechanism of action of abiraterone (CYP17 inhibition) means that the synthesis of both androgens and oestrogens is decreased. Therefore, there was the potential for clinical activity of abiraterone related to ER signalling pathway inhibition, independent of AR, in EOC. An adaptive design was utilised incorporating integral biomarker assessment to ensure that a sufficient number of patients with AR-positive EOC were included.

A key question is why more responses were not observed. We demonstrated that abiraterone substantially reduced oestradiol and testosterone levels and was therefore ‘hitting its target’. It is possible that, unlike prostate cancer, despite AR expression, the AR signalling pathway is not the most critical driver of tumour progression in recurrent EOC, and inhibition of this pathway alone is therefore insufficient to cause significant response. Nevertheless, the CBR rate of 26.2% at 12 weeks and more prolonged CBR at 6 months (11.9%) is clinically meaningful and should not be ignored in this population of patients where many have received several lines of therapy and have limited treatment options. There is evidence from a series of 29 paired pre- and post-chemotherapy EOC samples suggesting AR expression decreases with exposure to chemotherapy.^16^ The modest clinical activity may be due to prior treatment; 47.6% of CORAL patients had received three or more lines of prior therapy (median two lines). A limitation of the study was that biopsies were not mandatory at study entry or during the trial; no biopsies were received from patients with evaluable archival material preventing interrogation of the hypothesis that a fall in AR expression following multiple lines of therapy explained the low response rate seen.

Grade 3 hypertension (28.6%) and hypokalaemia (9.5%) were more common in CORAL than most other reported abiraterone studies.^25,26^ Grade 3 hypertension was noted in 20% of patients in the abiraterone-treated group (compared with 10% in the placebo group) within the LATITIUDE prostate cancer trial.^24^ A total of 57.1% of the patients who developed grade 3 hypertension were hypertensive pre-treatment. All instances of grade 3 hypokalaemia resolved following a short treatment interruption. Hypertension and hypokalaemia result from mineralocorticoid excess secondary to CYP17 inhibition by abiraterone. In one breast cancer study, the rate of grade 3 or 4 hypokalaemia was slightly higher (14%) than in CORAL.^27^

Beyond the licensed indication of abiraterone in prostate cancer, studies of abiraterone in breast cancer have been reported and the response rates have been modest.^26–28^ The results of these studies suggest that AR is not likely to be the main driver in endocrine-resistant or triple-negative breast cancer.

It is also important to consider potential mechanisms of abiraterone resistance. In prostate cancer, glucocorticoid-induced activation of mutated AR has been demonstrated, which could be overcome by increased abiraterone exposure or combining abiraterone with enzalutamide.^28^ Preclinical studies have shown that abiraterone directly activates the ER leading to breast cancer cell proliferation. This can be blocked by ER antagonists (e.g. fulvestrant).^29^ The combination of CYP17 inhibition (abiraterone) with drugs such as enzalutamide as an AR antagonist or fulvestrant (ER antagonist) may be worth investigating in ovarian cancer.

In addition to abiraterone, there are other agents that target the AR signalling pathway *via* other mechanisms of action. Enzalutamide inhibits androgens binding to AR, AR nuclear translocation and AR-mediated DNA binding. Tumour growth inhibition has been reported with enzalutamide in an EOC xenograft model.^21^ There is a phase II trial of enzalutamide in patients with AR+ (5% cut off) EOC who have had a maximum of three prior lines of cytotoxic therapy. The results of this trial will add to the CORAL study findings regarding the significance of AR and whether there is a role for the use of other AR targeting drugs in EOC.

The number of responses observed in this unselected population of patients with advanced EOC did not meet the threshold for continuation of the study. However, further studies of AR pathway targeted agents may be worth exploring in selected women with the low grade serous subtype. There may also be merit in utilising gene expression signatures. For example, preliminary results indicate that the PREDICT AR test (gene expression profiling) was significantly associated with OS in triple-negative breast cancer patients treated with enzalutamide.^30^ Correct patient selection will be key if AR targeted therapy is to have a future in the management of EOC.

## Supplemental Material

sj-docx-3-tam-10.1177_1758835920975352 – Supplemental material for Abiraterone in patients with recurrent epithelial ovarian cancer: principal results of the phase II Cancer of the Ovary Abiraterone (CORAL) trial (CRUK – A16037) Click here for additional data file. Supplemental material, sj-docx-3-tam-10.1177_1758835920975352 for Abiraterone in patients with recurrent epithelial ovarian cancer: principal results of the phase II Cancer of the Ovary Abiraterone (CORAL) trial (CRUK – A16037) by Susana Banerjee, Holly Tovey, Rebecca Bowen, Elizabeth Folkerd, Lucy Kilburn, Jennifer McLachlan, Marcia Hall, Nina Tunariu, Ayoma Attygalle, Joao Paulo Da Silveira Nogueira Lima, Sophie Perry, Peter Chatfield, Margaret Hills, Stan Kaye, Gert Attard, Mitch Dowsett and Judith M. Bliss in Therapeutic Advances in Medical Oncology

sj-pdf-4-tam-10.1177_1758835920975352 – Supplemental material for Abiraterone in patients with recurrent epithelial ovarian cancer: principal results of the phase II Cancer of the Ovary Abiraterone (CORAL) trial (CRUK – A16037) Click here for additional data file. Supplemental material, sj-pdf-4-tam-10.1177_1758835920975352 for Abiraterone in patients with recurrent epithelial ovarian cancer: principal results of the phase II Cancer of the Ovary Abiraterone (CORAL) trial (CRUK – A16037) by Susana Banerjee, Holly Tovey, Rebecca Bowen, Elizabeth Folkerd, Lucy Kilburn, Jennifer McLachlan, Marcia Hall, Nina Tunariu, Ayoma Attygalle, Joao Paulo Da Silveira Nogueira Lima, Sophie Perry, Peter Chatfield, Margaret Hills, Stan Kaye, Gert Attard, Mitch Dowsett and Judith M. Bliss in Therapeutic Advances in Medical Oncology

sj-tif-1-tam-10.1177_1758835920975352 – Supplemental material for Abiraterone in patients with recurrent epithelial ovarian cancer: principal results of the phase II Cancer of the Ovary Abiraterone (CORAL) trial (CRUK – A16037) Click here for additional data file. Supplemental material, sj-tif-1-tam-10.1177_1758835920975352 for Abiraterone in patients with recurrent epithelial ovarian cancer: principal results of the phase II Cancer of the Ovary Abiraterone (CORAL) trial (CRUK – A16037) by Susana Banerjee, Holly Tovey, Rebecca Bowen, Elizabeth Folkerd, Lucy Kilburn, Jennifer McLachlan, Marcia Hall, Nina Tunariu, Ayoma Attygalle, Joao Paulo Da Silveira Nogueira Lima, Sophie Perry, Peter Chatfield, Margaret Hills, Stan Kaye, Gert Attard, Mitch Dowsett and Judith M. Bliss in Therapeutic Advances in Medical Oncology

sj-tif-2-tam-10.1177_1758835920975352 – Supplemental material for Abiraterone in patients with recurrent epithelial ovarian cancer: principal results of the phase II Cancer of the Ovary Abiraterone (CORAL) trial (CRUK – A16037) Click here for additional data file. Supplemental material, sj-tif-2-tam-10.1177_1758835920975352 for Abiraterone in patients with recurrent epithelial ovarian cancer: principal results of the phase II Cancer of the Ovary Abiraterone (CORAL) trial (CRUK – A16037) by Susana Banerjee, Holly Tovey, Rebecca Bowen, Elizabeth Folkerd, Lucy Kilburn, Jennifer McLachlan, Marcia Hall, Nina Tunariu, Ayoma Attygalle, Joao Paulo Da Silveira Nogueira Lima, Sophie Perry, Peter Chatfield, Margaret Hills, Stan Kaye, Gert Attard, Mitch Dowsett and Judith M. Bliss in Therapeutic Advances in Medical Oncology
