# Supplementary material for: Abiraterone in patients with recurrent epithelial ovarian cancer: principal results of the phase II Cancer of the Ovary Abiraterone (CORAL) trial (CRUK – A16037)
Source: Ther Adv Med Oncol. 2020 Dec 29;12:1758835920975352. doi: 10.1177/1758835920975352 (PMC8013695; doi:10.1177/1758835920975352)
Supplement: sj-docx-3-tam-10.1177_1758835920975352 – Supplemental material for Abiraterone in patients with recurrent epithelial ovarian cancer: principal results of the phase II Cancer of the Ovary Abiraterone (CORAL) trial (CRUK – A16037) [file sj-docx-3-tam-10.1177_1758835920975352.docx]

**Supplementary Table S1.** Clinical benefit rate by subgroups

|  | **N** | **N. clinical benefit** | **CBR (95% CI)** | **p-value** |
| --- | --- | --- | --- | --- |
| AR+ | 29 | 8 | 27.6 (12.7 – 47.2) | 0.696 |
| AR- | 11 | 2 | 18.2 (2.3 – 51.8) |  |
|  |  |  |  |  |
| ER+ | 35 | 9 | 25.7 (12.5 – 43.3) | 0.473 |
| ER- | 2 | 1 | 50.0 (1.3 – 98.7) |  |
|  |  |  |  |  |
| PgR+ | 25 | 7 | 28.0 (12.1 – 49.4) | 1.000 |
| PgR- | 12 | 3 | 25.0 (5.5 – 57.2) |  |
|  |  |  |  |  |
| High grade | 37 | 8 | 21.6 (9.8 – 38.2) | 0.103 |
| Other | 5 | 3 | 60.0 (14.7 – 94.7)) |  |
|  |  |  |  |  |
| ≤2 prior lines | 22 | 6 | 27.3 (10.7 – 50.2) | 0.344 |
| 3 prior lines | 10 | 4 | 40.0 (12.2 – 73.8) |  |
| ≥4 prior lines | 10 | 1 | 10.0 (0.2 – 44.5) |  |

p-value is derived from Fisher’s exact test.
