# Supplementary material for: Abiraterone in patients with recurrent epithelial ovarian cancer: principal results of the phase II Cancer of the Ovary Abiraterone (CORAL) trial (CRUK – A16037)
Source: Ther Adv Med Oncol. 2020 Dec 29;12:1758835920975352. doi: 10.1177/1758835920975352 (PMC8013695; doi:10.1177/1758835920975352)
Supplement: sj-pdf-4-tam-10.1177_1758835920975352 – Supplemental material for Abiraterone in patients with recurrent epithelial ovarian cancer: principal results of the phase II Cancer of the Ovary Abiraterone (CORAL) trial (CRUK – A16037) [file sj-pdf-4-tam-10.1177_1758835920975352.pdf]

CORAL Supplementary appendix

1. Trial management

CORAL was approved by the Medicines and Healthcare products Regulatory Authority (MHRA) and the London - Westminster Research Ethics Committee (REC 13/LO/1599). The trial was endorsed by Cancer Research UK (CRUKE/12/052) and co-sponsored by The Institute of Cancer Research and Royal Marsden Hospital NHS Foundation Trust.

2. Methods

2.1. Eligibility Criteria

Inclusion Criteria

1. Histologically or cytologically confirmed epithelial ovarian, fallopian tube (FT) or primary peritoneal (PP) cancer and have progressed (radiological or CA125 criteria) within 12 months of last systemic anti-cancer therapy

2. Life expectancy of at least 12 weeks

3. Post-menopausal defined as:

   - Aged ≥ 18 years having had bilateral salpingo-oophorectomy (BSO)
   - Aged ≥ 45 years with intact uterus and amenorrhoeic for at least 12 months
   - FSH >40 U/L in patients who have had a hysterectomy and ovaries are intact (i.e. not had bilateral oophorectomy)

   Documentation is required for patients who have undergone irreversible surgical sterilisation by hysterectomy, bilateral oophorectomy or bilateral salpingectomy

4. ECOG performance status of 0-2

5. No prior hormone therapy (e.g. tamoxifen, aromatase inhibitor, progestogens, anti-androgens)

6. At least one line of prior platinum-based chemotherapy

7. Measurable or evaluable disease (if not measurable by RECIST v1.1 criteria, patients must be evaluable by GCIG CA125 criteria). See Appendix 2 for criteria

8. Archival primary tumour tissue (FFPE or 8-10 unstained slides) must be available. Otherwise, a biopsy must be carried out to obtain sufficient tissue for histological assessment

9. Haematological and biochemical indices within the ranges shown below.

Laboratory Test	Value required
Haemoglobin (Hb)	≥ 9.0 g/dL
Absolute neutrophil count	≥ 1.5 x 10^9/L
Platelet count	≥ 100 x 10^9/L
Serum potassium	≥3.5mmol/L
Serum bilirubin	≤ 1.5 x upper limit of normal (ULN)

Alanine aminotransferase (ALT) or aspartate aminotransferase (AST)	≤ 2.5 x (ULN)
Alkaline phosphatase (ALP)	< 5 x ULN
Creatinine Clearance<br><br>OR<br><br>Serum creatinine	≥ 50 mL/min (uncorrected value, calculated as per local policy)<br><br><1.5 x ULN

10. Aged 18 years or over
11. Written (signed and dated) informed consent and be capable of co-operating with treatment and follow-up

Exclusion Criteria

1. Tumours of mucinous, clear cell, malignant mixed mesodermal (MMMT) or non-epithelial ovarian cancers (e.g. Brenner tumours, Sex-cord tumours)
2. Radiotherapy (except for palliative reasons) or chemotherapy within the preceding three weeks (four weeks for investigational agent or within five half-lives of the investigational agent, whichever is longer)
3. Persistent grade 2 or greater toxicities from any cause except for alopecia or grade 2 peripheral neuropathy
4. Known leptomeningeal involvement or brain metastases
5. Clinical and/or biochemical evidence of hyperaldosteronism or hypopituitarism
6. Unresolved bowel obstruction or symptoms of sub-acute bowel obstruction
7. Major surgery within four weeks prior to commencement of trial treatment
8. Treatment with warfarin. Patients on warfarin for DVT/PE can be converted to LMWH at least one week prior to commencement of trial treatment
9. At high medical risk, as deemed by the Investigator, because of non-malignant systemic disease including active uncontrolled infection
10. Ascites on clinical examination or significant ascites present on baseline imaging (Note: patients that have ascitic drainage prior to study entry are eligible)
11. Known to be serologically positive for hepatitis B and/or hepatitis C
12. Active or uncontrolled autoimmune disease that may require corticosteroid therapy
13. History of clinically significant heart disease, e.g. myocardial infarction or arterial thrombotic event within six months, severe or unstable angina, or New York Heart Association Class III or IV heart disease
14. Systolic blood pressure >160 mm Hg and diastolic blood pressure >95 mm Hg documented on at least two different occasions
    [Note: Hypertension controlled by antihypertensive therapy is permitted].
15. Any other active malignancy requiring treatment
16. Patients for whom treatment with prednisone or prednisolone is contraindicated
17. Patients participating in or planning to participate in another interventional clinical trial. Participation in an observational trial is acceptable
18. **Any other condition which, in the Investigator's opinion, would not make the patient a good candidate for the clinical trial.**

2.2. Laboratory studies

The following antibodies were used for the laboratory studies:

Androgen receptor: DAKO M3562 Antibody clone AR441

Ki67: DAKO M7240 Antibody clone Mib-1

ER: LEICA/NOVOCASTRA Antibody clone 6F11

PGR: LEICA/NOVOCASTRA Antibody clone 16

HER2: DAKO K5207 HercepTest for HER2 protein over expression.
