# Supplementary figures and images for: Abiraterone in patients with recurrent epithelial ovarian cancer: principal results of the phase II Cancer of the Ovary Abiraterone (CORAL) trial (CRUK – A16037)
Source: Ther Adv Med Oncol. 2020 Dec 29;12:1758835920975352. doi: 10.1177/1758835920975352 (PMC8013695; doi:10.1177/1758835920975352)

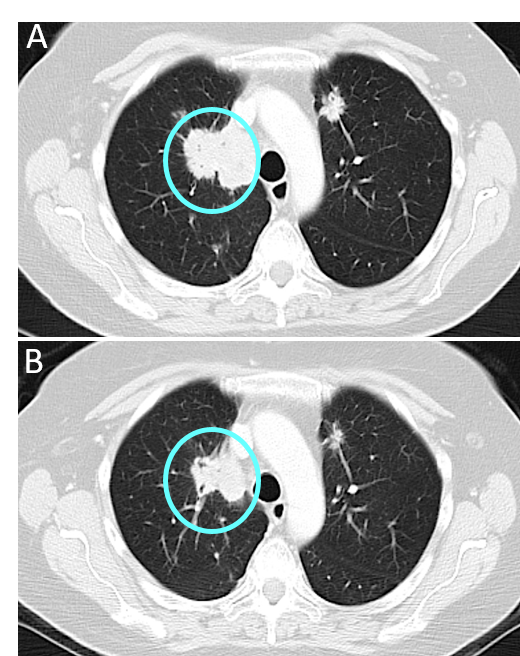

Supplement: sj-tif-1-tam-10.1177_1758835920975352 – Supplemental material for Abiraterone in patients with recurrent epithelial ovarian cancer: principal results of the phase II Cancer of the Ovary Abiraterone (CORAL) trial (CRUK – A16037) [file sj-tif-1-tam-10.1177_1758835920975352.tif]

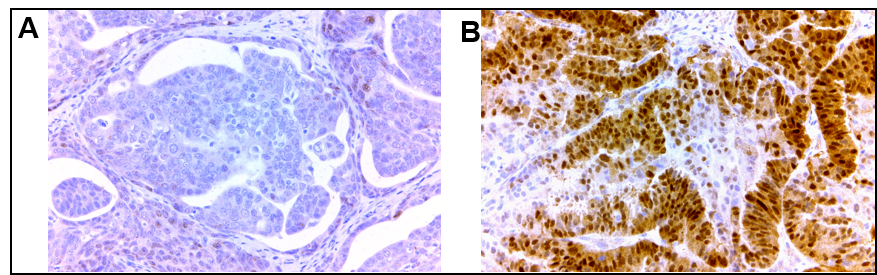

Supplement: sj-tif-2-tam-10.1177_1758835920975352 – Supplemental material for Abiraterone in patients with recurrent epithelial ovarian cancer: principal results of the phase II Cancer of the Ovary Abiraterone (CORAL) trial (CRUK – A16037) [file sj-tif-2-tam-10.1177_1758835920975352.tif]
